# Beneficial Effects of Adiponectin on Periodontal Ligament Cells under Normal and Regenerative Conditions

**DOI:** 10.1155/2014/796565

**Published:** 2014-07-13

**Authors:** Marjan Nokhbehsaim, Sema Keser, Andressa Vilas Boas Nogueira, Joni Augusto Cirelli, Søren Jepsen, Andreas Jäger, Sigrun Eick, James Deschner

**Affiliations:** ^1^Experimental Dento-Maxillo-Facial Medicine, University of Bonn, 53111 Bonn, Germany; ^2^Clinical Research Unit 208, University of Bonn, 53111 Bonn, Germany; ^3^Department of Diagnosis and Surgery, School of Dentistry, UNESP, 14801-903 Araraquara, SP, Brazil; ^4^Department of Periodontology, Operative and Preventive Dentistry, University of Bonn, 53111 Bonn, Germany; ^5^Department of Orthodontics, University of Bonn, 53111 Bonn, Germany; ^6^Department of Periodontology, Laboratory of Oral Microbiology, University of Bern, 3010 Bern, Switzerland

## Abstract

Type 2 diabetes and obesity are increasing worldwide and linked to periodontitis, a chronic disease which is characterized by the irreversible destruction of the tooth-supporting tissues, that is, periodontium. The mechanisms underlying the association of diabetes mellitus and obesity with periodontal destruction and compromised periodontal healing are not well understood, but decreased plasma levels of adiponectin, as found in diabetic and obese individuals, might be a critical mechanistic link. The aim of this in vitro study was to examine the effects of adiponectin on periodontal ligament (PDL) cells under normal and regenerative conditions, and to study the regulation of adiponectin and its receptors in these cells. Adiponectin stimulated significantly the expression of growth factors and extracellular matrix, proliferation, and in vitro wound healing, reduced significantly the constitutive tumor necrosis factor-*α* expression, and caused a significant upregulation of its own expression. The beneficial actions of enamel matrix derivative on a number of PDL cell functions critical for periodontal regeneration were partially enhanced by adiponectin. The periodontopathogen *Porphyromonas gingivalis* inhibited the adiponectin expression and stimulated the expression of its receptors. In conclusion, reduced levels of adiponectin, as found in type 2 diabetes and obesity, may compromise periodontal health and healing.

## 1. Introduction

The prevalence of diabetes mellitus and its associated comorbidities is increasing worldwide [1]. Obesity is considered a major risk factor for type 2 diabetes and has also risen substantially throughout the globe over the past decades [2, 3]. A number of meta-analyses have demonstrated that diabetes mellitus and obesity are linked to periodontitis, a chronic disease characterized by the irreversible destruction of the tooth-supporting tissues, that is, periodontium [4–7]. The periodontium consists of the gingiva, periodontal ligament (PDL), root cementum, and alveolar bone. Microorganisms, such as* Fusobacterium nucleatum, Aggregatibacter actinomycetemcomitans, Porphyromonas gingivalis*, and* Treponema denticola*, in the subgingival plaque on the tooth surfaces are essential for the initiation and progression of periodontitis [8, 9]. Several cofactors, such as smoking, can increase the risk for periodontitis [10]. The periodontopathogenic microorganisms provoke an inflammatory host response, which involves inflammatory mediators, such as interleukin- (IL-) 1*β*, IL-6, IL-8, tumor necrosis factor (TNF) *α*, and cyclooxygenase (COX) 2 in the periodontal tissues. If the periodontal inflammation is exaggerated and sustained, matrix degradation, bone resorption, periodontal pocket formation, and even tooth loss can follow [10, 11].

Periodontitis can be successfully treated by either nonsurgical or surgical approaches, sometimes combined with antibiotics. The main goal of periodontal therapy is to arrest inflammation and periodontal tissue destruction by reducing or even eliminating the periodontopathogenic microorganisms in the periodontal pockets [12]. In order to promote regenerative healing, bioactive molecules, such as enamel matrix derivative (EMD), are applied during periodontal surgery [13–15]. EMD stimulates periodontal cells to produce growth factors, such as transforming growth factor (TGF) *β*1 and vascular endothelial growth factor (VEGF), matrix molecules, such as periostin (POSTN), and osteogenesis-related factors, such as runt-related transcription factor (RUNX) 2. Moreover, EMD has been shown to accelerate the in vitro wound healing [16, 17]. The beneficial effects of EMD on periodontal regeneration are accomplished, at least in part, by bone morphogenetic protein (BMP) and TGF*β*, which trigger SMAD (sma- and mad-related protein) and non-SMAD signaling cascades [18–22]. Interestingly, microbial, inflammatory, and biomechanical signals can interfere with the regeneration-promotive effects of EMD on periodontal cells, which highlights the critical role of the cell microenvironment for regenerative healing [23–25].

Interestingly, diabetes mellitus and obesity are associated not only with the initiation and progression of periodontitis, but also with compromised healing after periodontal therapy [26–28]. The mechanisms underlying the associations of diabetes mellitus and obesity with periodontitis and compromised periodontal healing are not well understood so far. However, altered plasma levels of adipokines, as found in diabetic and obese individuals, might be a critical mechanistic link in these associations. Adipokines are bioactive molecules, which are produced in the adipose tissue. In diabetes mellitus and obesity, plasma levels of proinflammatory adipokines, such as visfatin, leptin, and resistin, are increased, whereas the levels of adiponectin, which is an anti-inflammatory adipokine, are decreased. The imbalance between pro- and anti-inflammatory adipokines contributes to the low-grad inflammatory state, as observed in diabetes mellitus and obesity [29–32].

Adiponectin is mainly synthesized by adipocytes. By binding to its receptors (AdipoR1 and AdipoR2), adiponectin activates the adenosine monophosphate-activated protein kinase (AMPK) and other pathways [33–35]. Like other adipokines, adiponectin has both metabolic and immune functions. Adiponectin enhances fatty acid oxidation, insulin sensitivity, and glucose uptake and inhibits the hepatic gluconeogenesis. In addition, it exerts anti-inflammatory effects [33, 36–39]. We have previously shown that adiponectin abrogated the proinflammatory actions of lipopolysaccharide (LPS) from* P. gingivalis* on gingival epithelial cells, suggesting that adiponectin might be protective in the periodontium [40]. Interestingly, adiponectin has also been suggested to support regeneration in various tissues, such as bone and muscle [41]. However, in certain cells, conditions, and diseases, such as rheumatoid arthritis, adiponectin levels are increased and associated with proinflammatory effects [42]. As shown in our previous studies, proinflammatory signals can reduce the regenerative capacity of periodontal cells and therefore inhibit regenerative healing [43–46]. So far, little is known about the actions of adiponectin on periodontal homeostasis and regeneration. The aim of this in vitro study was to examine the effects of adiponectin on periodontal cells under normal and regenerative conditions and to study the regulation of adiponectin and its receptors in these cells.

## 2. Materials and Methods

### 2.1. Culture and Stimulation of Cells

PDL cells were obtained from 18 periodontally healthy donors, who underwent tooth extractions for orthodontic reasons. Written informed consent and approval of the Ethics Committee of the University of Bonn were obtained. PDL cells (passages 3–5) were seeded on culture plates (50,000 cells/well) and grown to 80% confluence in Dulbecco's minimal essential medium (DMEM, Invitrogen, Karlsruhe, Germany) supplemented with 10% fetal bovine serum (FBS, Invitrogen), 100 units penicillin, and 100 *μ*g/mL streptomycin (Invitrogen) at 37°C in a humidified atmosphere of 5% CO_2_. Before the experiments, the FBS concentration was reduced to 1%, and medium was changed every other day.

In order study the effects of adiponectin on PDL cells, physiological concentrations of adiponectin (0.3, 1, and 3 *μ*g/mL; HMW oligomers, R&D Systems, Minneapolis, MN, USA) were added to the cells [47]. Cells were also treated with EMD (Emdogain, Straumann, Freiburg, Germany) used to mimic regenerative conditions at a concentration of 100 *μ*g/mL. To simulate inflammatory conditions, cells were incubated with IL-1*β* (1 ng/mL; Calbiochem, San Diego, CA, USA). An infectious environment was mimicked by treating cells with the inactivated oral periodontopathogens* F. nucleatum* ATCC 25586 (optical density: 0.1),* A. actinomycetemcomitans* ATCC 43718 (OD: 0.1),* P. gingivalis* ATCC 33277 (OD: 0.025–0.2), or* T. denticola* ATCC 35405 (OD: 0.1). Bacteria were suspended in PBS (OD_660 _nm = 1, equivalent to 1.2 × 10^9^ bacterial cells/mL) and subjected two times to ultrasonication (160 W for 15 min) resulting in a complete killing. In order to ensure that results were comparable, EMD, IL-1*β*, and the periodontopathogens were applied at the same physiological doses as in our previous experiments [43–46, 48–50].

In order to unravel the intracellular signaling involved in possible actions of adiponectin, PDL cells were also preincubated with a specific inhibitor against the AMPK signaling pathway (dorsomorphin; 5 *μ*M; Calbiochem, San Diego, CA, USA) 45 min before the experiments.

### 2.2. Measurement of Wound Closure

As previously described, an established in vitro wound healing model was used to analyze the effects of adiponectin on the wound closure in PDL cell cultures [43–46]. Briefly, cells were grown until confluence and 3 mm wide wounds, that is, cell-free areas, were created in the cell monolayers. The wounded cell monolayers were then exposed to adiponectin (3 *μ*g/mL) in the presence and absence of EMD and documented by inverse microscopy (Axiovert 25 C, 5x objective, Carl Zeiss, Oberkochen, Germany) and digital photography (Kodak DC 290, Kodak, Stuttgart, Germany) over 4 d. Afterwards, the wound widths were measured and the wound closure was determined with special software (Alpha DigiDoc 1000, Alpha Innotech, San Leandro, CA, USA).

### 2.3. Analysis of Gene Expression

RNA was extracted by using an RNA extraction kit (Qiagen, Hilden, Germany), and a total of 1 *μ*g of RNA was reverse transcribed using iScript Select cDNA Synthesis Kit (Bio-Rad Laboratories, Munich, Germany) at 42°C for 90 min followed by 85°C for 5 min. Expression of TGF*β*1, VEGF, POSTN, RUNX2, IL-6, IL-8, tumor necrosis factor (TNF) *α*, cyclooxygenase (COX) 2, Ki67, adiponectin, adiponectin receptors (AdipoR1 and AdipoR2), and glyceraldehyde-3-phosphate dehydrogenase (GAPDH) was determined by real-time PCR using the iCycler iQ detection system (Bio-Rad Laboratories), SYBR Green (Bio-Rad Laboratories), and specific primers (QuantiTect Primer Assay, Qiagen). One *μ*L of cDNA was amplified as a template in a 25 *μ*L reaction mixture containing 12.5 *μ*L 2x QuantiFast SYBR Green PCR Master Mix (Qiagen), 2.5 *μ*L of primers, and 9 *μ*L deionized water. The mixture was heated initially at 95°C for 5 min and then followed by 40 cycles with denaturation at 95°C for 10 s and combined annealing/extension at 60°C for 30 s. Data were analyzed by the comparative threshold cycle method.

### 2.4. Measurement of Protein Levels

Protein levels of active TGF*β*1, VEGF and adiponectin in the supernatants of PDL cells were determined by commercially available enzyme-linked immunosorbent assay (ELISA) kits (for TGF*β*1 and VEGF from R&D Systems, for adiponectin from RayBiotech, Norcross, GA, USA) according to the manufacturer's instructions. The absorbance was measured by using a microplate reader (PowerWave x, BioTek Instruments, Winooski, VT, USA) at 450 nm. Data were normalized by cell number.

### 2.5. Analysis of SMAD1/5/8 Nuclear Translocation

PDL cells were fixed with 4% paraformaldehyde in PBS pH 7.4 for 10 min, washed with PBS (Sigma-Aldrich, Munich, Germany), and treated with 0.1% Triton X-100 (Sigma-Aldrich) for 5 min, as previously reported [45, 46]. Then, cells were washed again and blocked with nonfat dry milk (Bio-Rad Laboratories) for 1 h. After washing, cells were incubated with a primary rabbit anti-SMAD1/5/8 antibody (1 : 200; Santa Cruz Biotechnology, Santa Cruz, CA, Germany) for 90 min and with CY3-conjugated goat anti-rabbit IgG (1 : 2,000; Abcam, Cambridge, MA, USA) for 45 min. The SMAD1/5/8 nuclear translocation was analyzed using an Axioplan 2 imaging microscope (20x objective; Carl Zeiss). Images were captured with a PVCAM camera and the VisiView capturing software (Visitron Systems, Puchheim, Germany).

### 2.6. Statistical Analysis

All experiments were performed in triplicate and repeated at least twice. Mean values and standard errors of the mean (SEM) were calculated. Parametric (ANOVA followed by Dunnett's or Tukey's tests) and nonparametric (Mann-Whitney *U*) tests were applied for statistical analysis by using the IBM SPSS Statistics 22 software (IBM Corporation, Armonk, NY, USA). Differences between groups were considered significant at *P* < 0.05.

## 3. Results

### 3.1. Effects of Adiponectin on Growth Factors, Matrix Molecules, and Inflammatory Mediators

**Figure 1:**
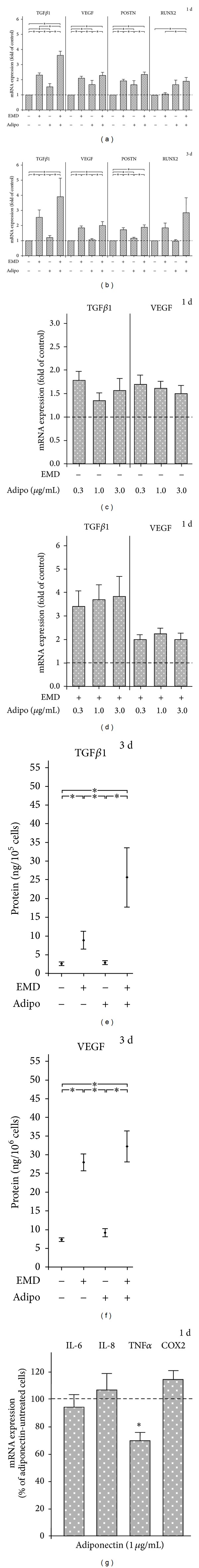
Effects of EMD and/or adiponectin (1 *μ*g/mL) on the mRNA expression of TGF*β*1, VEGF, POSTN, and RUNX2 at 1 d (a) and 3 d (b). Untreated cells were used as control. Mean ± SEM (*n* = 18); ∗significant (*P* < 0.05) difference between groups. Effects of various concentrations of adiponectin (0.3, 1, and 3 *μ*g/mL) on the mRNA expression of TGF*β*1 and VEGF in the absence (c) and presence (d) of EMD at 1 d. Mean ± SEM (*n* = 9). Effects of EMD and/or adiponectin (1 *μ*g/mL) on TGF*β*1 (e) and VEGF (f) protein levels at 3 d. Unstimulated cells were used as control. Mean ± SEM (*n* = 18); ∗significant (*P* < 0.05) difference between groups. Effects of adiponectin (1 *μ*g/mL) on the IL-6, IL-8, TNF*α*, and COX2 mRNA expressions at 1 d (g). Unstimulated cells were used as control. Mean ± SEM (*n* = 15); ∗significantly (*P* < 0.05) different from control.

First we examined possible effects of adiponectin on the synthesis of molecules known to be involved in periodontal destruction and/or regeneration. As shown in Figure 1(a), adiponectin caused a significant upregulation of the TGF*β*1, VEGF, and POSTN mRNA expressions at 1 d. A pronounced but insignificant upregulation was also observed for RUNX2 in adiponectin-treated cells at this time point. Adiponectin also led to a slight but significant increase in the POSTN mRNA expression at 3 d (Figure 1(b)). Interestingly, preincubation of cells with a specific inhibitor against the AMPK pathway inhibited the adiponectin-induced mRNA expression of TGF*β*1, VEGF, POSTN, and RUNX2 by 62.85 ± 1.13%, 5.79 ± 1.89%, 42.27 ± 3.01, and 28.40 ± 2.17%, respectively, at 1 d.

As expected from previous studies [44–46], EMD stimulated significantly the TGF*β*1, VEGF, and POSTN mRNA expressions at 1 d and 3 d (Figures 1(a) and 1(b)). EMD also enhanced the RUNX2 mRNA expression at 3 d, but this stimulatory effect was not significant (Figure 1(b)). Next we wondered whether adiponectin would interfere with the beneficial actions of EMD on PDL cells. At 1 d, adiponectin increased significantly the EMD-induced mRNA expression of TGF*β*1 and RUNX2, as demonstrated in Figure 1(a). Adiponectin had similar effects on both molecules at 3 d, but the effects did not reach significance (Figure 1(b)). Further experiments revealed that the effects of adiponectin on TGF*β*1 and VEGF mRNA expressions were also observed at lower and higher adiponectin concentrations (Figures 1(c) and 1(d)). No significant differences were observed between cells exposed to different adiponectin concentrations (Figures 1(c) and 1(d)). The upregulation of TGF*β*1 and VEGF by EMD and the further increase of the EMD-induced TGF*β*1 mRNA expression were also observed at protein level (Figures 1(e) and 1(f)).

Finally, we studied whether adiponectin exerts regulatory effects on the mRNA expression of proinflammatory mediators. As depicted in Figure 1(g), adiponectin caused a significant inhibition of the constitutive TNF*α* mRNA expression at 1 d, whereas the IL-6, IL-8, and COX2 mRNA expressions were not significantly regulated by this adipokine.

### 3.2. Effects of Adiponectin on In Vitro Wound Healing and Proliferation

**Figure 2:**
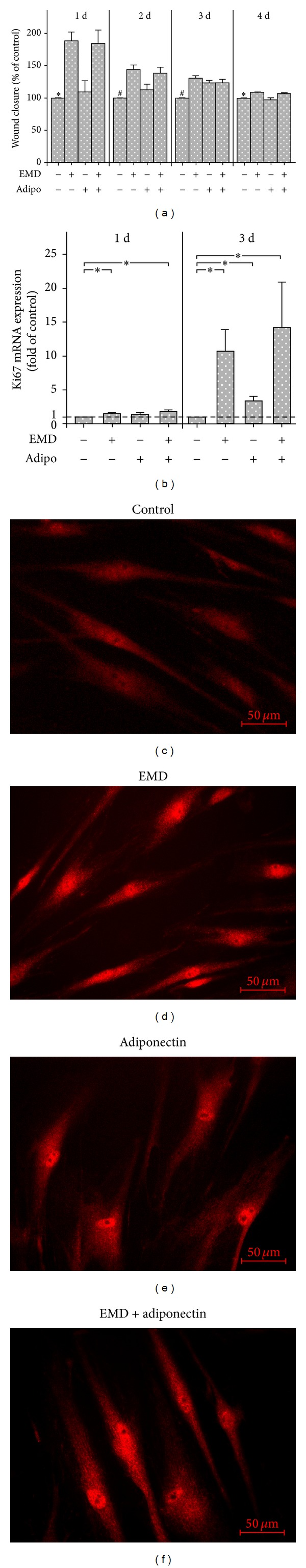
Effects of EMD and/or adiponectin (3 *μ*g/mL) on wound closure over 4 d (a). Untreated cells were used as control. Mean ± SEM (*n* = 12); ∗significantly (*P* < 0.05) different from cells treated with EMD, either alone or with adiponectin; ^#^significantly (*P* < 0.05) different from all other groups. Effects of EMD and/or adiponectin (1 *μ*g/mL) on the mRNA expression of Ki67 at 1 d and 3 d (b). Untreated cells were used as control. Mean ± SEM (*n* = 18); ∗significant (*P* < 0.05) difference between groups. Effects of EMD on SMAD1/5/8 nuclear translocation in the presence and absence of adiponectin (1 *μ*g/mL) at 60 min, as analyzed by immunofluorescence (c–f). Untreated cells served as control. Experiments were performed in triplicate and repeated twice. Images from one representative donor are shown.

Next we examined the effects of adiponectin on the in vitro wound fill rate. In the absence of EMD, adiponectin accelerated significantly the wound closure by 13.37 ± 8.63% and 23.96 ± 3.78, at 2 d and 3 d, respectively. However, no regulatory effects of adiponectin on the wound closure were observed in the presence of EMD (Figure 2(a)). Since the wound fill rate depends on cell proliferation, we then analyzed the actions of adiponectin on Ki67, a marker of proliferation, in the presence and absence of EMD. As shown in Figure 2(b), EMD increased significantly the Ki67 mRNA expression at 1 d and 3 d. In the absence of EMD, adiponectin also caused a significant Ki67 upregulation at 3 d. However, no significant effects of adiponectin on the Ki67 mRNA expression were observed in the presence of EMD at 1 d and 3 d (Figure 2(b)).

### 3.3. Actions of Adiponectin on SMAD1/5/8 Signaling

Since EMD has been shown to trigger SMAD signaling, we also examined the effects of adiponectin on the SMAD1/5/8 nuclear translocation in the presence and absence of EMD. As expected [45, 46], EMD induced the SMAD1/5/8 nuclear translocation at 60 min (Figures 2(c) and 2(d)). Similarly to EMD, adiponectin also activated the SMAD1/5/8 signaling pathway (Figure 2(e)). Moreover, a pronounced nuclear accumulation of SMAD1/5/8 was also observed in cells, which were simultaneously exposed to EMD and adiponectin (Figure 2(f)).

### 3.4. Regulation of Adiponectin by Microbial and Inflammatory Signals

**Figure 3:**
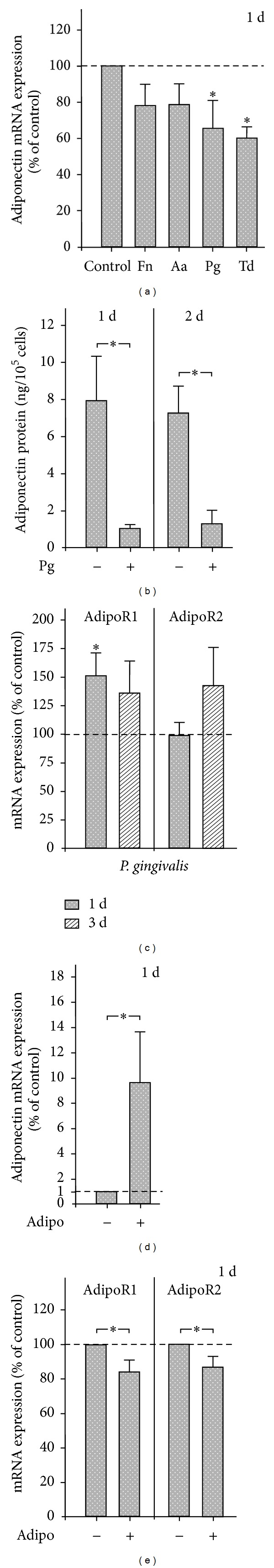
Effects of* F. nucleatum* (*Fn*),* A. actinomycetemcomitans* (*Aa*),* P. gingivalis* (*Pg*), and* T. denticola* (*Td*) (all OD: 0.1) on the mRNA expression of adiponectin in PDL cells at 1 d (a). Untreated cells were used as control. Mean ± SEM (*n* = 9); ∗significantly (*P* < 0.05) different from control. Effect of* P. gingivalis* (*Pg*; OD: 0.1) on the adiponectin protein level in medium from PDL cells at 1 d and 2 d (b). Untreated cells were used as control. Mean ± SEM (*n* = 9); ∗significant (*P* < 0.05) difference between groups. Effects of* P. gingivalis* (OD: 0.1) on the mRNA expression of adiponectin receptors (AdipoR1 and AdipoR2) at 1 d and 3 d (c). Untreated cells were used as control. Mean ± SEM (*n* = 15); ∗significantly (*P* < 0.05) different from control. Effects of adiponectin (1 *μ*g/mL) on its own mRNA expression (d) and the expression of its receptors AdipoR1 and AdipoR2 (e) at 1 d. Untreated cells served as control. Mean ± SEM (*n* = 18); ∗significant (*P* < 0.05) difference between groups.

Next we analyzed whether the constitutive mRNA expression of adiponectin and its receptors is affected by periodontopathogens and inflammatory mediators. Whereas* F. nucleatum* and* A. actinomycetemcomitans* had no significant effects,* P. gingivalis* and* T. denticola* diminished significantly the mRNA expression of adiponectin at 1 d (Figure 3(a)). Further experiments revealed that the* P. gingivalis*-induced inhibition of the adiponectin mRNA expression was dose-dependent, with the strongest inhibition at the highest dose (data not shown).* P. gingivalis* also inhibited significantly the release of adiponectin protein from PDL cells at 1 d and 2 d, as analyzed by ELISA (Figure 3(b)). Moreover,* P. gingivalis* increased significantly the mRNA expression of AdipoR1 at 1 d (Figure 3(c)). In addition,* P. gingivalis* enhanced the AdipoR2 mRNA expression at 1 d and 3 d, but these stimulatory effects did not reach significance (Figure 3(c)). Cells were also exposed to IL-1*β*, but this proinflammatory cytokine had no significant effects on the mRNA expression of adiponectin and its receptors at 1 d and 3 d (data not shown).

### 3.5. Regulation of Adiponectin and Its Receptors by Adiponectin

Finally, we analyzed the autostimulatory effect of adiponectin and the actions of this adipokine on its receptors. As shown in Figures 3(d) and 3(e), adiponectin caused a very pronounced and significant upregulation of its own mRNA expression and a slight but significant downregulation of the AdipoR1 and AdipoR2 mRNA expressions at 1 d.

## 4. Discussion

The present study shows that adiponectin is capable of stimulating the expression of growth factors and extracellular matrix, in vitro wound healing, and proliferation in PDL cells. Our findings also demonstrate for the first time that the actions of EMD on a number of PDL cell functions critical for periodontal regeneration are not abrogated by adiponectin, which is in contrast to proinflammatory adipokines, such as visfatin and leptin, as previously shown [45, 46]. In our experiments, adiponectin even upregulated the EMD-induced expression of growth and osteogenesis-associated factors. Therefore, reduced levels of adiponectin, as found in type 2 diabetes and obesity, may compromise periodontal health and healing and represent a pathomechanistic link between periodontal and systemic diseases [51–54].

Adiponectin caused an upregulation of TGF*β*1, VEGF, POSTN, and RUNX2, which underlines the beneficial role of adiponectin in periodontal homeostasis as well as soft and hard tissue healing. VEGF supports wound healing by stimulation of vascular permeability and leukocyte recruitment, survival, proliferation, migration, and invasion of endothelial cells, as well as modulation of bone remodeling [55–57]. TGF*β*, another growth factor, stimulates wound healing by its activating effects on migration, chemotaxis, and proliferation of monocytes/macrophages, fibroblasts, and endothelial cells, keratinocyte migration and reepithelialization, extracellular matrix production, and stem cell differentiation [58–60]. POSTN supports formation of high stiffness collagen through effective collagen cross-linking. This matrix molecule helps disperse mechanical forces applied to the PDL and plays a role in osteoblast adhesion, differentiation, and survival [61–63]. RUNX2 is a critical transcription factor in osteoblast commitment and differentiation and, thereby, bone formation [64]. In the present study, adiponectin caused an upregulation of RUNX2, even though the effect was not significant. These findings concur with observations by other investigators, who have also found an upregulated RUNX2 expression in addition to an enhanced ALP expression and mineralization in adiponectin-treated PDL cells [65]. An adiponectin-induced stimulation of mineralization has also been shown in dental pulp cells [66]. Interestingly, adiponectin can also inhibit osteoclastogenesis, which indicates that adiponectin not only promotes bone formation, but also protects against bone resorption [67, 68]. Therefore, these observations suggest that adiponectin could play a role in periodontal and/or peri-implant treatment strategies [69].

The results of our study also revealed that the stimulatory effects of adiponectin on TGF*β*1, VEGF, POSTN, and RUNX2 are dependent on the AMPK pathway and that adiponectin can trigger SMAD1/5/8 signaling in PDL cells. Furthermore, adiponectin did not inhibit the actions of EMD on the nuclear translocation of SMADs, which is in contrast to the inhibitory effects of visfatin and leptin, as previously shown [45, 46]. Whether other pathways are involved in the actions of adiponectin on PDL cells should be examined in further studies.

Adiponectin also accelerated the wound closure in an established in vitro wound healing assay. In this assay, the wound closure depends, at least in part, on cell proliferation. Interestingly, adiponectin stimulated significantly the PDL cell proliferation, which is in accordance with findings by other investigators. Future studies should clarify if and how adiponectin impacts on migration, which also determines the wound closure in this healing assay.

Since it has been reported that adiponectin can exert pro- and anti-inflammatory effects, we also studied the regulatory actions of adiponectin on the expression of inflammatory mediators [39, 42]. Adiponectin reduced significantly the constitutive TNF*α* expression. A number of studies have shown that adiponectin counteracts the stimulatory effects of periodontopathogens or IL-1*β* on the expression of proinflammatory cytokines in periodontal cells [40, 65, 70]. Therefore, our findings are in line with these studies and emphasize the anti-inflammatory characteristics of this adipokine.

Another novel finding of our study is the observation that adiponectin is expressed in PDL cells and that it exerts an autostimulatory effect on its expression. As previously reported, AdipoR1 and AdipoR2 are expressed in PDL cells [65, 71, 72]. Our experiments confirm the expression of both adiponectin receptors in PDL cells. Moreover, periodontopathogens, such as* P. gingivalis*, were capable of inhibiting the adiponectin expression and stimulating the expression of its receptors. These novel observations are in accordance with our previous study, which has demonstrated a downregulation of adiponectin and an upregulation of its receptors in gingival biopsies from periodontally diseased subjects as compared to periodontally healthy individuals [48]. Our findings also concur with the clinical observations that plasma adiponectin levels are decreased in periodontitis and increased again after periodontal therapy [47, 73–75]. Future in vivo studies should examine the adiponectin synthesis by PDL cells in a more complex environment and clarify whether PDL cells can directly affect circulating adiponectin levels. A mouse model of experimental periodontitis might be useful for this purpose.

The concentrations of adiponectin used in our experiments are in the physiological range, as measured in the gingival crevicular fluid (GCF) [47]. As in our previous studies, cells were exposed to IL-1*β*, which was used to mimic inflammatory conditions in vitro [43, 46, 48, 50]. This proinflammatory cytokine is increased in GCF and gingival tissues at inflamed sites [76–78]. PDL cells were also treated with suspensions of* F. nucleatum, A. actinomycetemcomitans, P. gingivalis*, and* T. denticola*, as in previous experiments [46, 48–50]. These bacteria are strongly linked to periodontitis and were therefore used to simulate microbial conditions in vitro [79–84]. Nevertheless, periodontitis is caused by not only these few bacteria, but also a complex bacterial biofilm. Therefore, further studies should examine the effects of other periodontitis-associated microorganisms on the expression of adiponectin and its receptors in periodontal cells. The bacterial suspensions contained disrupted cell wall particles with a high amount of LPS, but additional microbial components may have been present in the suspensions. If these components can also regulate the expression of adiponectin and its receptors should be examined in further studies.

In summary, the present study shows that adiponectin stimulates the expression of growth factors and extracellular matrix, in vitro wound healing, and proliferation. Furthermore, our data also demonstrate for the first time that the actions of EMD on a number of PDL cell functions critical for periodontal regeneration are not inhibited but rather enhanced by adiponectin. In conclusion, reduced levels of adiponectin, as found in type 2 diabetes and obesity, may compromise periodontal health and healing.
